# The RNA chaperone activity of the *Trypanosoma brucei* editosome raises the dynamic of bound pre-mRNAs

**DOI:** 10.1038/srep19309

**Published:** 2016-01-19

**Authors:** W.-Matthias Leeder, Christin Voigt, Michael Brecht, H. Ulrich Göringer

**Affiliations:** 1 Molecular Genetics, Darmstadt University of Technology, Schnittspahnstraße 10, 64287 Darmstadt, Germany

## Abstract

Mitochondrial transcript maturation in African trypanosomes requires an RNA editing reaction that is characterized by the insertion and deletion of U-nucleotides into otherwise non-functional mRNAs. The reaction is catalyzed by editosomes and requires guide (g)RNAs as templates. Recent data demonstrate that the binding of pre-edited mRNAs to editosomes is followed by a chaperone-type RNA remodeling reaction. Here we map the changes in RNA folding using selective 2′-hydroxyl acylation analyzed by primer extension (SHAPE). We demonstrate that pre-mRNAs in their free state adopt intricately folded, highly stable 2D-structures. Editosome binding renders the pre-mRNAs to adopt 2D-conformations of reduced stabilities. On average about 30% of the nucleotides in every pre-mRNA are affected with a prevalence for U-nucleotides. The data demonstrate that the chaperone activity acts by increasing the flexibility of U-residues to lower their base-pairing probability. This results in a simplified RNA folding landscape with a reduced energy barrier to facilitate the binding of gRNAs. The data provide a first rational for the enigmatic U-specificity of the editing reaction.

RNA chaperones catalyze the formation of the thermodynamically most stable RNA conformation by lowering the energetic barriers between misfolded RNA populations and by mediating the unwinding and refolding of non-functional RNA conformations^1^,^2^,^3^,^4^. As a consequence of that function, cells express a whole gamut of RNA chaperones, ATP-dependent RNA helicases and RNA annealing factors, which in effect contribute to all levels of gene expression, ribonucleoprotein assembly and RNA-mediated regulation. Within the different processes some RNA chaperones act as accessory factors, while others are integral components of the macromolecular machineries that catalyze the different reaction pathways^5^. Mitochondrial transcript maturation in African trypanosomes requires an RNA editing reaction in which non-functional pre-mRNAs are converted into translatable mRNAs by the site-specific insertion and deletion of exclusively U-nucleotides^6^. Some transcripts are edited by the insertion of literally hundreds of U’s, which ultimately account for more than 50% of the mRNA sequence. The reaction is catalyzed by a 0.8 MDa mitochondrial multienzyme complex termed the editosome^7^. RNA editing has been shown to involve RNA annealing factors^8^ as well as DEAD-box-type RNA helicases^9^,^10^,^11^, however, recent evidence suggests that the editosome also executes an RNA chaperone function^12^. Although the molecular details of the chaperone reaction are not understood, it has been demonstrated that it is capable of refolding pre-edited mRNAs^12^, which are characterized by an unusual nucleotide bias. Especially all substantially *i.e.* pan-edited pre-mRNAs are extremely purine-rich with in some cases purine/pyrimidine (R/Y) ratios >2.5 (Supplementary Table 1).

By monitoring the local dynamic of >7200 nucleotides in several pre-edited mRNAs in their free and editosome-bound folding states we uncovered that the RNA chaperone activity of the *T. brucei* editosome acts by raising the dynamic of bound substrate RNAs. The reaction shows a preponderance for U-nucleotides and thus provides a first rational for the inexplicable U-specificity of the RNA editing reaction.

## Results

### Pre-edited mRNAs adopt thermodynamically highly stable 2D-structures

**Figure 1:**
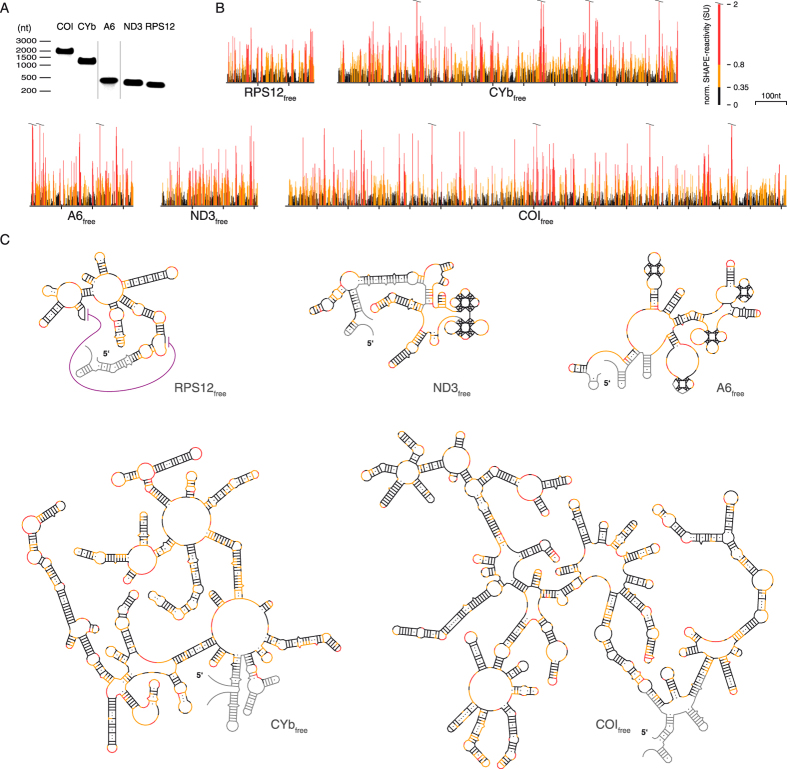
SHAPE-derived 2D-structures of *T. brucei* mitochondrial pre-mRNAs in their free folding states. (**A**) Gel electrophoretic characterization of the *T. brucei* COI-, CYb-, A6-, ND3- and RPS12-transcripts. (**B**) Normalized SHAPE-reactivity profiles of all 5 transcripts as free RNAs. Black: low (<0.35SU); yellow: medium (0.35 ≤ SU < 0.8); red: high (≥0.8SU) normalized SHAPE-reactivities. SU: SHAPE-unit. nt: nucleotides. A representative example of the individual steps to generate normalized SHAPE-profiles is given in Supplementary Fig. 2. (**C**) SHAPE-derived MFE-2D-structures of the 5 transcripts in their free folding states (coloring scheme as above). Purple line: pseudoknot fold in the RPS12-transcript. Grey: plasmid-derived 5′- and 3′-nt extensions or no data.

To map changes in the structural landscape of pre-edited mRNAs upon binding to editosomes we used the SHAPE (selective 2′-hydroxyl acylation analyzed by primer extension) chemical probing method developed by Weeks *et al.*^13^,^14^,^15^. SHAPE provides quantitative structural information with single-nucleotide resolution based on the accessibility of 2′-OH groups of conformationally flexible ribonucleotides to electrophilic acylation reagents. For the analysis we used 5 mitochondrial transcripts: the pre-mRNA of ribosomal protein S12 (RPS12), the transcript of subunit 3 of the NADH dehydrogenase (ND3), the pre-mRNAs of subunit 6 of the mitochondrial ATPase (A6) and of apocytochrome b (CYb) and the transcript of cytochrome oxidase I (COI). The different RNAs range in size from 282 nucleotides to 1647 nucleotides and include pre-mRNAs that are pan-edited (A6, ND3, RPS12), only marginally edited (CYb) and never-edited (COI). All RNAs were generated by run off *in vitro* transcription from linearized plasmid DNA constructs (Fig. 1A) and were refolded at native pH and ion conditions. Structure probing was performed using the electrophile 1-methyl-7-nitroisatoic anhydride (1M7). 1M7 is a fast reacting compound with a half-life of 14 sec^16^. The acylation is self-limiting due to hydrolysis of the reagent and has been shown to be insensitive to solvent accessibility constraints^17^.

Figure 1B shows representative, normalized SHAPE-profiles for all 5 pre-mRNAs. Depending on the transcript up to 8 independent experiments were performed yielding Pearson correlation coefficients (*r*) of up to 0.87 ± 0.04 (Supplementary Fig. 1). Altogether >3600 SHAPE-data points were generated. The individual reactivities were converted into pseudo Gibbs free energy terms (ΔG_SHAPE_) to calculate experimentally derived minimal free energy (MFE) 2D-structures as shown in Fig. 1C. All pre-mRNAs adopt highly complex 2D-folds with ΔΔG’s of −823 kcal/mol (COI), −496 kcal/mol (CYb), −152 kcal/mol (RPS12), −137 kcal/mol (ND3) and −137 kcal/mol (A6). These values calculate to ΔG/nt-values between −0.34 kcal/mol and −0.48 kcal/mol and ΔG/bp-values between −1.27 kcal/mol and −1.59 kcal/mol (Table 1). In order to verify these values we performed temperature-dependent UV-hyperchromicity measurements and compared the measured hyperchromicities to the expected values of the SHAPE-derived 2D-structures (Supplementary Fig. 3). On average the two data sets differ only by 15% and thus corroborate the complex folds of the different transcripts. Importantly, nearest neighbor-based 2D-folding predictions^18^,^19^ underestimate the experimentally derived thermodynamic stabilities on average by 30% (Table 1) emphasizing the need for structure probing experiments. Almost all known RNA 2D-structure features can be found in the 5 transcripts including a high number of G/U basepairs and a pseudoknot in the pre-mRNA of RPS12 (summarized in Supplementary Table 2).

### Pre-edited A6 and ND3 transcripts contain multiple GQ-elements

**Figure 2:**
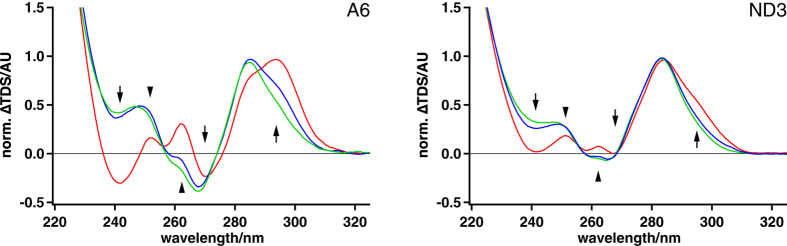
Experimental verification of GQ-folds - thermal difference (TD) spectra. Normalized double-difference TD-spectra of the A6- and ND3-transcript in the presence of KCl (red), NaCl (blue) and LiCl (green). Signature peaks of GQ-folds are marked by arrows^23^. Additional differences are indicated by arrowheads. AU: arbitrary unit. Peak intensities decrease in the presence of GQ-destabilizing Na^+^- and Li^+^-ions.

**Figure 3:**
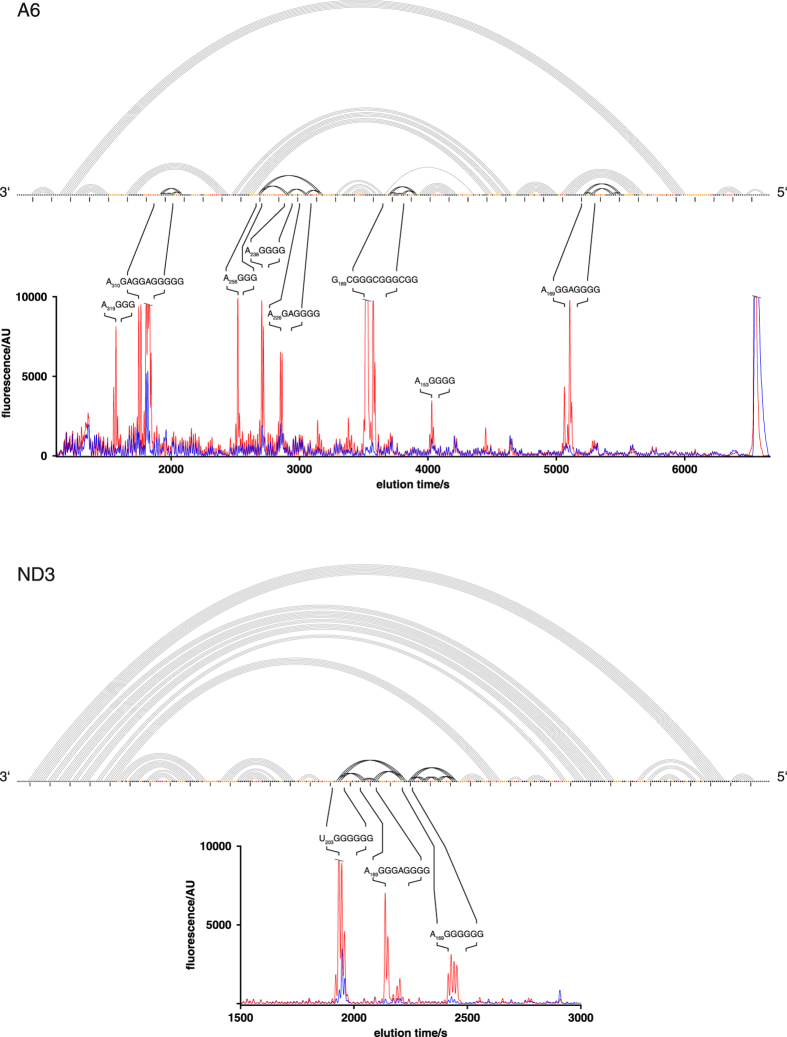
Experimental verification of GQ-folds – RT-stop assays. Capillary electrophoretic (CE)-traces of premature reverse transcriptase (RT)-stop assays of the A6- and ND3-transcripts in the presence of GQ-stabilizing K^+^-ions (red) and GQ-destabilizing Na^+^-ions (blue). Sequence identities of the individual RT-stop signals are shown on top of the different peaks. RNA secondary structures are annotated as arcs above the CE-traces. AU: arbitrary unit.

One of the identified RNA folding features in the different pre-edited transcripts is of special interest: In two of the pre-mRNAs we identified multiple G-quadruplex (GQ)-folds^20^,^21^. GQ-elements represents the most stable structures in DNA and RNA. The 2D-structure of the A6-transcript contains four and the ND3-transcript contains two GQ-folds (Fig. 1C). In order to derive an additional experimental verification of the GQ-elements we measured UV-thermal difference spectra (TDS)^22^,^23^. TD-spectra represent the arithmetic difference between a high- and low-temperature UV-absorbance spectrum *i.e* between RNA in its unfolded and fully folded states. In the presence of stabilizing K^+^-ions, GQ-folds have been shown to display a set of signature peaks (243 nm, 273 nm and 295 nm)^23^ and Fig. 2 shows representative TD-spectra for both, the A6 and ND3 transcripts. The two pre-mRNAs are characterized by all GQ-specific minima and maxima, which disappear in the presence of destabilizing Na^+^- and Li^+^-ions. The exact positions of the different GQ’s were identified in premature reverse transcriptase (RT) termination assays^24^,^25^. As shown in Fig. 3, at 75 mM K^+^ all G-nucleotide containing sequences involved in the formation of the individual GQ’s were identified as strong RT-stop signals.

### Editosome binding increases the dynamic of pre-edited mRNAs

**Figure 4:**
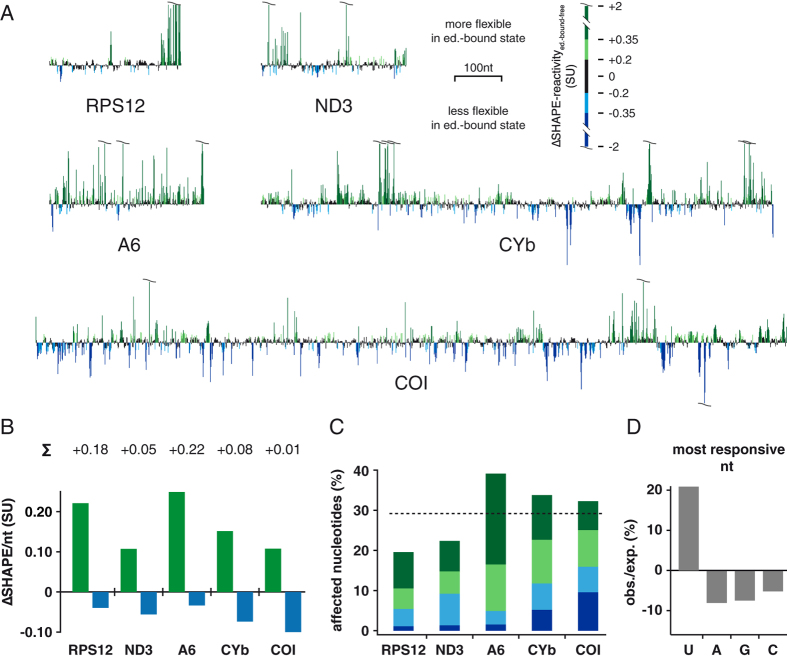
SHAPE-reactivity changes in the editosome-bound folding state. (**A**) Difference (Δ) SHAPE-reactivity profiles of the RPS12-, ND3-, A6-, CYb- and COI-transcripts. Green: nt-positions with increased SHAPE-reactivities in the editosome-bound folding state (dark green: +0.35 ≤ SU ≤  +2; light green: +0.2 ≤ SU <  +0.35). Blue: nt-positions with decreased SHAPE-reactivities in the editosome-bound folding state (light blue: −0.2 ≥ SU > −0.35; dark blue: −0.35 ≥ SU ≥ −2. Black: non-responsive nt-positions (−0.2 ≤ SU ≤  +0.2). (**B**) Bar graph summing up all increased (green) and decreased (blue) ΔSHAPE-reactivities. Numbers represent the difference of the two values and demonstrate a general increase in nt-flexibility upon editosome binding. SU: SHAPE-unit. nt: nucleotides. (**C**) Bar graph illustrating the percentage of affected nucleotides in all 5 transcripts. Stippled line: Arithmetic mean. (**D**) Bar graph illustrating the under-/over-representation of SHAPE-responsive nucleotides in the editosome-bound folding state. Values were calculated by determining the fraction of all 4 ribonucleotides (A, G, C, U) within the top 10% of responsive positions in the 5 transcripts divided by the relative abundance of each nt in that interval.

Next we probed the structures of the selected pre-mRNAs in their editosome-bound folding state. For that we preincubated the transcripts with active editosomes in a 1:1 molar stoichiometry. Binding equilibrium was achieved at 27 °C for 45–60 min. Per RNA between 3 and 7 independent SHAPE-experiments were performed ultimately generating 3614 data points (*r* up to 0.81 ± 0.07; Supplementary Fig. 1). As before, normalized SHAPE-reactivity profiles were plotted (Supplementary Fig. 4) to calculate experimentally-derived MFE-2D-structures for the 5 pre-mRNAs in their editosome-bound folding state. Importantly, in their bound form, all transcripts adopt less stable 2D-folds with on average 15% reduced Gibbs free energies (ΔΔG’s) (Table 1). Based on the specificity of the acylation reagent this indicates a change in the folding landscape to increase the flexibility of the different pre-mRNAs. Figure 4A shows ΔSHAPE-profiles of all pre-mRNAs highlighting the reactivity differences (increases/decreases) between the two folding states (free *vs.* editosome-bound). In spite of the different sizes of the transcripts the data demonstrate a global increase in reactivity especially for all edited transcripts. Reactivity differences generally spread throughout the entire primary sequences with individual nucleotides showing hyperreactive characteristics (>|2SU|)^17^. A quantitative comparison of the reactivity differences is given in Fig. 4B. While the never-edited COI transcript is only marginally affected (0.01SU), all edited transcripts (RPS12, A6, ND3, CYb) show net reactivity increases between 0.05-0.22SU. The same picture can be drawn from the arithmetic mean (AM) of the normalized SHAPE-reactivities (Supplementary Fig. 5). On average 29% of the nucleotide positions in every pre-mRNA are affected in the conformational transition (Fig. 4C). Furthermore, by analyzing the correlation between nucleotide identity and changes in SHAPE-reactivity, we identified that U-nucleotides represent the most responsive nucleotides in the folding transition (Supplementary Fig. 6). From a total of 93 nucleotide positions that undergo a conversion from <0.35SU to >0.8SU 66 nucleotides or 71% are U-residues. Considering the nt-bias in the different pre-mRNAs and by plotting the ratio of observed/expected SHAPE-reactivities, the U-specificity of the chaperone activity becomes strikingly obvious. (Fig. 4D). This demonstrates that the RNA folding landscape of all 5 transcripts is simplified by increasing the flexibility of primarily U’s.

**Figure 5:**
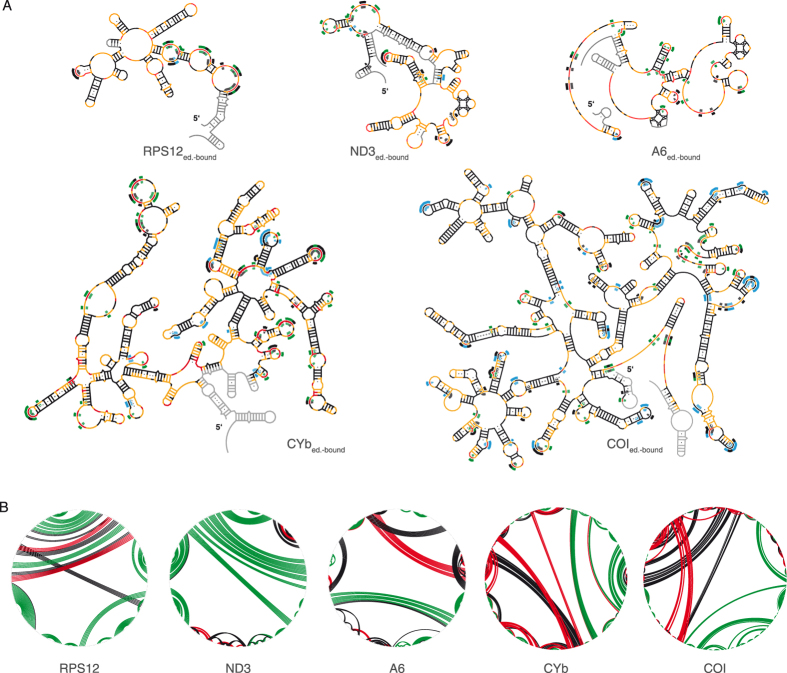
Mapping the structural transition from free RNA to editosome-bound RNA. (**A**) SHAPE-derived MFE-2D-structures of all 5 transcripts in their editosome-bound conformation. U-nt within the top 10% of responsive nt-positions are shown in green (increased SHAPE-reactivity) and blue (decreased SHAPE-reactivity). Black: non-uridine responsive nt. (**B**) Circle plot representations of the SHAPE-derived 2D-structures of the 5 pre-mRNAs. Base pairs are shown as colored lines. Green: basepairs present in both RNA folding states. Red: basepairs unique to the editosome-bound RNA conformations. Black: basepairs unique to the free RNA folding states. Plots were generated using CircleCompare of RNAstructure^26^.

Figure 5A shows the SHAPE-derived MFE-2D-structures of the 5 RNAs in their editosome-bound form highlighting the 10% most responsive nucleotide positions. Compared to the free RNA folding states the average ΔG/nt-value of the 5 transcripts drops from −0.41 kcal/mol to −0.33 kcal/mol and the mean ΔG/bp-value decreases from −1.43 kcal/mol to −1.34 kcal/mol (Table 1). Two GQ-elements in the A6 transcript and one GQ-fold in the ND3 transcript no longer exist, demonstrating that the editosome chaperone activity possesses GQ-resolving potency analogous to the RecQ and Pif1-families of helicases^20^. Similarly, the pseudoknot in the RPS12 transcript is absent in the edititosome-bound folding state. Figure 5B illustrates the 2D-folds and structural changes in a “circle plot” representation^26^. On average 65% of all basepairs in the free RNAs are preserved in the editosome-bound conformations. Roughly 20% of the basepairs are exclusive to either of the two folding states and the basepairing-content of all editosome-bound RNA structures is invariably lower than in the free RNA folding state (maximally 10%).

### The editosome-bound folding state favors the formation of pre-mRNA/gRNA hybrid RNAs

**Figure 6:**
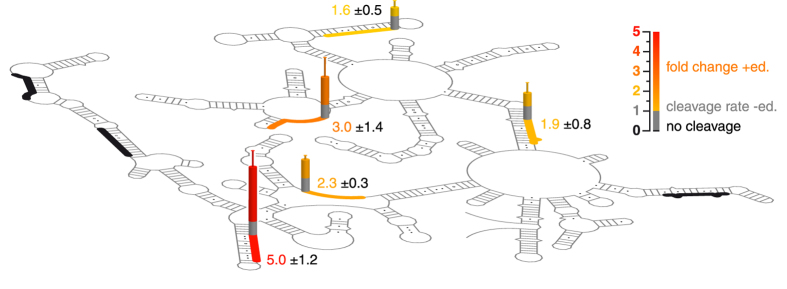
Guide RNA/pre-mRNA-hybrid formation in the free and editosome-bound RNA folding states. Background: SHAPE-derived 2D-fold of the CYb pre-mRNA. The binding sites of eight base-complementary DNA-oligonucleotides acting as quasi gRNAs are shown as thick lines. Colored bars indicate the change in RNaseH-based cleavage of the different DNA/pre-mRNA-hybrid molecules in the free and editosome-bound RNA folding states. Grey: RNaseH-cleavage in the free RNA folding state (=1). Yellow-red: >1–5-fold stimulus in the editosome-bound folding state. Black: no RNaseH cleavage. Errors are standard deviations (SD). A representative example of the data acquisition, normalization and quantification procedures is shown in Supplementary Fig. 8.

The mechanistic rational for increasing the flexibility of editosome-bound RNAs is to overcome the structural rigidity of the different pre-edited transcripts to lower the energy barrier for subsequent steps in the editing cycle. Based on our current knowledge, the next step in the reaction is the annealing of a gRNA molecule to generate a pre-mRNA/gRNA hybrid, which defines the pre-mRNA endonucleolytic cleavage site for the U-insertion/deletion reaction. As a consequence of the data above, the formation of gRNA/pre-mRNA hybrid RNAs should be thermodynamically favored if the pre-mRNA is in its “flexible” *i.e.* editosome-bound state and disfavored if the transcript is in its “rigid” (free) configuration. To experimentally falsify this hypothesis we developed an assay in which the formation of pre-mRNA/gRNA hybrid is mimicked by short DNA-oligonucleotides as “guiding” molecules. This enabled us to quantitatively assess hybrid formation by RNaseH cleavage. Representative results are shown in Fig. 6 using CYb as a target pre-mRNA. Invariably, hybrid formation is favored if the transcript is in the editosome-bound folding state and not in the free RNA conformation. Depending on the guiding DNA-oligonucleotide up to 5-fold differences were measured between the two folding states, which demonstrates that at least one function of the editosome chaperone activity is to alter the pre-mRNA folding landscape to promote the formation of gRNA/pre-mRNA hybrid RNAs.

## Discussion

Mitochondrial gene expression in African trypanosomes requires RNA editing. By inserting and deleting only U-nucleotides, the processing reaction converts non-functional, pre-edited transcripts into translation-competent mRNAs. Editing follows a multistep catalytic reaction cycle that is mediated by a mitochondria-specific, high molecular mass protein particle known as the editosome^7^. Editosomes are characterized by a single RNA interaction domain on their surface^12^ and the binding of pre-edited mRNAs as substrates in the process has been shown to involve a chaperone-type RNA refolding reaction^12^. Unfortunately, the biochemical and structural details of the chaperoning process are not understood. Here we aimed at characterizing the editosome-inherent RNA chaperone activity by monitoring the folding transitions of 5 pre-edited mRNAs from their free folding state to their editosome-bound conformation using site-specific chemical probing^13^,^14^,^15^. We demonstrate that the pre-edited mRNAs in their free state adopt strikingly elaborate RNA folding characteristics with thermodynamic stabilities resembling structural RNAs. Two of the tested transcripts (A6, ND3) even contain multiple, up to 4 G-quadruplex elements, which represent the most stable folding motifs in DNA and RNA^20^. However, editosome binding changes the structures of the different transcripts by simplifying their folding landscapes through an increase in RNA dynamic. About 30% of the nucleotide positions in every RNA are involved in the folding transition and invariably, the resulting editosome-bound MFE-structures have reduced thermodynamic stabilities. Although the different RNAs are not completely unfolded at the tested 1:1 editosome/pre-mRNA stoichiometry, fifty percent of the GQ-structures no longer exist in the editosome-bound RNA conformation. Importantly, the increase in RNA dynamic has a direct consequence for the editing reaction cycle and thus provides a rational for the chaperone function. Rate limiting step of the reaction is the binding of gRNA molecules to form short, intermolecular gRNA/pre-mRNA hybrid structures. These hybrid elements define the endonucleolytic cleavage sites within the pre-mRNA molecules, which represents the first step in the reaction cycle. The formation of gRNA/pre-mRNA hybrid RNAs has been shown to involve matchmaking-type RNA/RNA annealing factors^8^, however, the data above demonstrate that the editosome itself contributes to the formation of hybrid RNAs. By increasing the dynamic of bound pre-mRNAs, the activity raises the probability for a gRNA to anneal to its base-complementary target site, thereby initiating the reaction cycle.

Interestingly, the RNA folding landscape of the pre-edited mRNAs is altered by specifically increasing the dynamic of preferentially U-nucleotides in the different transcripts. This nucleotide specificity finds precedence in the RNA chaperone mechanism of the retroviral NC and hnRNP A1 proteins, which have been shown to execute a G-centric refolding activity^4^. U-nucleotides represent promiscuous base-pairing partners^27^. They form homo- and hetero-basepairs with all other ribonucleotides and thus we propose that the U-specificity is a consequence of a selection process to globally lower the basepairing probability of pre-mRNAs by raising the dynamic of predominantly U’s. This assures that the highest number of possible basepairs is affected. As a consequence, it is tempting to speculate that this might represent the driving force for the enigmatic U-specificity of the RNA editing process as a whole. The unfolding reaction should thermodynamically and perhaps kinetically “accelerate” as the editing reaction proceeds since the U-content dramatically increases (especially for all pan-edited mRNAs). Furthermore, the U-centricity might also explain why editing involves roughly 10-times more U-insertions than U-deletions^6^.

The data also suggest that the editosome does not discriminate between folded and misfolded RNAs or between transcripts that undergo editing or are never edited. COI-RNA as a never-edited transcript, behaved qualitatively and quantitatively identical to all other RNAs. This indicates that the difference between a productive and non-productive chaperone reaction is simply the probability increase in forming a gRNA/pre-mRNA hybrid, which drives the reaction towards the next step in the editing cycle. Which of the protein components of the editosome executes the chaperone activity is not known to date. It has been suggested that the activity resides within the different OB-fold proteins of the complex^12^ based on the observation that the yeast Rrp44 polypeptide executes an OB-fold-driven RNA chaperone activity^28^,^29^. Importantly, since 1M7 does not probe solvent accessibility^17^ no conclusion as to the contact site(s) of the different transcripts with the editosome can be made. However, we predict that the catalytic complex displays a surface domain that is highly disordered and flexible^30^, which enables it to interact with a diverse landscape of RNAs of different length and 3D-conformations. This flexibility is likely central to the chaperone activity because it allows the editosome to remain in contact with RNA as it is remodeled and edited^3^,^31^.

This is the first time that a dynamic reaction step of the RNA editing reaction cycle has been demonstrated experimentally. The editosome amplifies the intrinsic dynamic behavior of bound substrate RNAs^32^ and induces multiple, U-specific RNA-equilibrium motions^33^,^34^,^35^ to counteract the high thermodynamic stabilities of pre-edited mRNAs. This U-nucleotide centricity provides a first physicochemical rational for the perplexing U-specificity of the editing reaction.

## Methods

### Cloning and RNA synthesis

Mitochondrial genes encoding subunit 6 of the mitochondrial ATPase (A6), apocytochrome b (CYb), NADH dehydrogenase subunit 3 (ND3), ribosomal protein S12 (RPS12) and cytochrome c oxidase subunit I (COI) were PCR-amplified from *T. brucei* Lister 427 genomic DNA^36^ using the following DNA-oligonucleotide primers (KpnI and SacI restriction endonuclease recognition/cleavage sites are underlined): A6_forw. GGGGTACC-AGAGGAATTTTGGGCGGAA-GAG; A6_rev. CCGAGCTCCTATAACTCCAAAATCACAACTTTC; COI_forw. GGGGTACCAT-GTTTTTTCTATGTCTTGTGTGC; COI_rev. CCGAGCTCTATAT-AAAAGAATAATAGGAAGG; CYb_forw. GGGGTACCAGCGGAGAAAAAAG-AAAGGGTC; CYb_rev. CCGAGCTCCTAATCTAACCTACACACTATG; ND3_forw. GGGGTACCTCAAAAAA-TCCTCGCCTTTTTACTTTAG; ND3_rev. CCGAGCTCCTTGATGTTAGTAT-AAATGATTATATG; RPS12_forw. GGGGTACCC-TAATACACTTTTGATAACAA-ACTAAAG; RPS12_rev. CCGAGCT-CCCTACCAAACATAAATGA-ACCTG. PCR-amplicons were cloned into the KpnI and SacI sites of phagemid pBS SK^-^ (Invitrogen) and transcripts were generated by run off *in vitro* transcription from linearized plasmids using T7-RNA polymerase. RNAs were purified from non-incorporated NTP’s by size exclusion chromatography, EtOH-precipitated and dissolved in 10 mMTris/HCl pH 7.5, 1 mM EDTA (TE).

### Editosome preparations

Editosomes were isolated from insect stage *Trypanosoma brucei* using the monomorphic strain Lister 427^36^. Cells were grown in SDM79-medium^37^ to a cell density of 1 × 10^7^ cells/mL. After harvesting cells were disrupted by N_2_-cavitation at isotonic conditions^38^. Mitochondrial vesicles were separated in Percoll step gradients and lysed in 1% (v/v) Nonidet P-40 in editing buffer (EB: 20 mM HEPES/KOH, pH 7.5, 30 mM KCl, 10 mM Mg(OAc)_2_, 1 mM DTT) containing 1 mM PMSF, 1 μg/mL leupeptin and 10 μg/mL trypsin inhibitor. The detergent lysates were further separated by isokinetic ultracentrifugation in 10–35% (v/v) linear glycerol gradients as in Göringer *et al.*, 1994^39^ and after fractionation, 20 S fractions were assayed for their RNA editing^40^,^41^ (Supplementary Fig. 7) and RNA binding activity^42^. RNA binding and RNA editing-competent fractions were stored at −20 °C.

### SHAPE-modification

The modification reagent 1-methyl-7-nitroisatoic anhydride (1M7) was synthesized as described^43^. Mitochondrial pre-mRNAs (0.1 μM) were denatured by heating to 95 °C (2 min) followed by snap cooling on ice. RNA refolding was achieved by equilibration in EB for 30 min at 27 °C, which represents the optimal growth temperature of insect-stage trypanosomes. RNA samples were split and treated either with 3.5 mM 1M7 in DMSO or the same volume of neat DMSO. Modification reactions were quenched after 70 sec by the addition of an equal volume of ddH_2_O. RNAs were recovered by EtOH precipitation and desalted by size exclusion chromatography. 1M7-modifications in presence of 20 S editosomes were performed at a molar pre-mRNA:editosome ratio of 1:1. Editosome/pre-mRNA complexes were incubated at 27 °C for 45–60 min and modified RNA was recovered by phenol extraction followed by EtOH precipitation and desalting by size exclusion chromatography.

### Reverse transcription and data processing

Equimolar amounts of fluorescently labeled DNA oligonucleotide primers (see below) were annealed to 1M7-modified or unmodified RNA samples in 0.25xTE pH 7.5 by heating to 95 °C (2 min), cooling to 50 °C (10 min) and snap cooling on ice. Reverse transcription was performed in 50 mM Tris/HCl pH 8.3, 75 mM KCl or NaCl (see below), 3 mM MgCl_2_, 2.5 mM DTT, 0.25 mM each dNTP and 0.75 U/μL RiboLock RNase inhibitor (Invitrogen). The reaction was started by prewarming the samples for 1.5 min prior to the addition of 5U/μL SuperScript III reverse transcriptase (RT, Invitrogen). The COI- and CYb-transcripts were incubated at 52 °C for 1 h. The pre-mRNA for RPS12 for 20 min at 40 °C and the ND3- and A6-transcripts for 5 min at 50 °C followed by 20 min at 55 °C. K^+^ was used for the COI- and CYb-RNAs and Na^+^ for the A6-, ND3- and RPS12-transcripts. Sequencing reactions were carried out using unmodified RNA, fluorescently labeled DNA oligonucleotide primer and ddNTPs at a final concentration of 0.125 mM each. The ddNTP identity was chosen depending on the transcript analyzed. Reverse transcription was stopped by snap cooling and the addition of 0.1 vol. 4 M NaOH followed by heating to 95 °C (5 min). Samples were pooled, EtOH precipitated and redissolved in HiDi formamid (ABI/Life technologies) for capillary electrophoresis. Raw electrophoretic traces were analyzed using SHAPEfinder^44^ utilizing the boxplot approach to determine the number of statistical outliers. Normalized SHAPE-reactivities were the result of averaging a minimum of 3 independent experiments. The following 5′-fluorescently labeled DNA-primer molecules were used: M13_reverse: 6-FAM/JOE/6-TAMRA-CAGGAAACAGCTA-TGACCATG; T3 reverse: 6-FAM/JOE/TAMRA-AATTAACCCTCACTAAAGG-GAAC; CYb_840-818: 6-FAM/JOE/6-TAMRA CATAAACTTATCTGGGATTGCC; CYb_603-581: 6-FAM/JOE/6-TAMRA-CCTATCACAAAATGC-ATCAGAAC; CYb_310-282: 6-FAM/JOE/6-TAMRA-TAAATCAT-ACTAATATATGTGTGTCAAAC; COI_1391-1367: 6-FAM/JOE/6-TAMRA-GTCAACAAAAGCAT-ACCATACAATG; COI_1144-1123: 6-FAM/JOE/6-TAMRA-CATAGTGGAAATGTGCAACAAC; COI_1004-978: 6-FAM/JOE/6-TAMRA-CACATATCTGTGAATAAGAAGCTATAG; COI_877-857: 6-FAM/JOE/6-TAMRA-GATG-CGCTCAAACAAACATAC; COI_619-602: 6-FAM/JOE/6-TAMRA-CAGCCAAAACGGGAAGTG; COI_479-453: 6-FAM/JOE/6-TAMRA-CTAG-AATACCAAGAAA-ATGCACAGAG.

### SHAPE-directed RNA folding

Normalized SHAPE-reactivities were used as pseudo Gibbs free energy values to guide the folding of the different pre-mRNAs using RNAstructure v5.6^19^,^45^. In addition, ShapeKnots^26^ was used to search for pseudoknots using normalized SHAPE-reactivities and the default parameters of ShapeKnots (p1 = 0.35 kcal/mol; p2 = 0.65 kcal/mol). To extrapolate the ∆G of a pseudoknot-containing minimum free energy (MFE) structure to 27 °C the ∆∆G of the MFE with and without the pseudoknot at 37 °C was added to the free energy of the MFE at 27 °C. G-quartet (GQ)-containing RNA structures were folded by initially forcing the corresponding sequence stretches into a single-stranded conformation. To estimate the ∆G of the complete structure, the free energy contribution of the GQ was calculated with the help of ViennaRNA v2.1.9^46^. In cases where the energy model of ViennaRNA was not applicable, the free energy of the competing, canonical 2D-structure was calculated using RNAstructure v5.6. Free energy contributions calculated by either method were finally added to the ∆G of the GQ-free structure. Non-canonical and canonical basepairs were introduced based on the primary data and isolated basepairs between highly reactive nt positions were removed from the final structures. Due to the unusual high distribution of SHAPE-reactivities in the A6-pre-mRNA in its editosome-bound state, box plot-normalized and averaged peak integrals corresponding to the modified reaction of both folding states were scaled against the invariable background followed by normalization to the outlier-revised free state^14^.

### Experimental verification of GQ-folds

GQ-structures were identified by premature RT-stop signals in the presence of GQ-stabilizing (K^+^) and GQ-destabilizing (Na^+^) ion conditions. Experiments were conducted analogous to the RT-SHAPE-procedure except that fluorescently labeled cDNAs were generated from unmodified transcripts in the presence of either 75 mM K^+^ or 75 mM Na^+^. cDNA fragments were resolved by capillary electrophoresis. Nucleotide identities were determined by cDNA-sequencing.

### Thermal difference spectra

Spectroscopic measurements were carried out in a thermoelectrically controlled UV-spectrophotometer in 0.5 mL quarts cuvettes. Prior to the analysis, RNAs were heat-denatured in 0.1 mL TE pH 7.5 (2 min) and snap cooled on ice before the addition of concentrated folding buffer. Equilibration was allowed for 15 min and RNA concentrations were set to an initial A_260_ = 1. TD spectra were recorded in 5 mM sodium cacodylate pH 6.8 at 27 °C and 95 °C from 325 nm to 220 nm with a scan rate of 250 nm/min generating 1 data point every 0.42 nm. Monovalent ions (Li^+^, Na^+^, K^+^) were titrated from 1 mM to 58 mM using concentrated stock solutions keeping dilution effects to a minimum. Samples were allowed to equilibrate for 10 min between each ion-titration step. TD spectra were generated by subtracting the normalized high temperature spectrum from the normalized low temperature spectrum. Double difference TD spectra were generated from the 1 mM and 58 mM cation titration spectra followed by normalization to the major peak around 290 nm. All spectra were smoothed by Savitzky-Golay filtering^47^.

### UV-melting curves

Pre-mRNA transcripts were dissolved in 0.5xTE pH 7.5 (50 μL), heated to 95 °C (2 min) and snap cooled on ice before the addition of concentrated folding buffer to yield a final volume of 0.5 mL and a final buffer concentration of 5 mM Na-cacodylate pH 6.8, 70 mM NaCl and 2 mM MgCl_2_. RNA concentrations were adjusted to A_260_ = 0.5. Denaturation/renaturation profiles were measured at 260 nm between 20 °C and 95 °C at a heating/cooling rate of 1 °C/min (data aquisition: 0.3 data points/°C). Fraction folded (α)-plots were generated from the the melting curves as: α(T) = a/(a + b); a, b: distances to the upper and lower baselines. Baselines were set by linear regression. Hyperchromicity values (A_rel_) were calculated by two different procedures. (1) “Nearest neighbor“ approach using equation: A_rel_ (%) = ((A_260_RNA_unfolded_/(A_260_ss + A_260_ds)) −1 × 100. Molar extinction coefficients of the fully unfolded RNAs, the double-stranded (ds) and the single-stranded (ss) subdomains of the SHAPE-derived 2D-structures were calculated using OligoCalc^48^ followed by a conversion to absorbance values. GQ-forming nucleotides were treated as ss and non-canonical bp were treated as Watson/Crick bp. (2) “Fractional approach” using equation: A_rel_ (%) = ((1/f_ss_ + f_ds_ × N + f_GQ_ × M) –1) × 100. M = 0.953 and N = 0.729 accounting for 37% hyperchromicity; f = fraction.

### RNaseH-based “gDNA” annealing assay

The ability of free and editosome-bound pre-mRNAs to form gRNA/pre-mRNA hybrid RNAs was analyzed using a panel of “guiding” (g)DNA oligonucleotides complementary to different regions of the CYb-transcript (see numbers in brackets). The following oligodeoxynucleotides were used: CYb-1 (150–161): ACAAAATATCAA; CYb-2 (227–238): GTAAATATAATA; CYb-3 (255–266): ATTGACTTAAAT; CYb-4 (459–470): TCACTTCCCCAA; CYb-5 (651–662): AACATATCTCTC; CYb-7 (840–851): AAAAACAAACCC; CYb-8 (967–978): ACTCATTCATAT; CYb-9 (1045–1056): CAAAAATAATAA. Annealed gDNA/pre-mRNA hybrid molecules were identified by RNaseH cleavage. For that, co-transcriptionally [^32^P]-labeled CYb-RNA (1 nM) was incubated with an eqimolar amount of editosomes and incubated for 15 min at 27 °C in EB. After the addition of 10–1000 nM DNA-oligonucleotide RNaseH cleavage was performed for 20 min at 27 °C using 0.01 U/μL *E. coli* RNaseH. RNA fragments were phenol-extracted followed by EtOH precipitation. Cleavage products were electrophoretically separated in 8 M urea-containing 5% (w/v) polyacrylamide gels, visualized by phosphorimaging and densitometrically quantified.

## Additional Information

**How to cite this article**: Leeder, W.-M. *et al.* The RNA chaperone activity of the *Trypanosoma brucei* editosome raises the dynamic of bound pre-mRNAs. *Sci. Rep.* **6**, 19309; doi: 10.1038/srep19309 (2016).

## Supplementary Material

Supplementary Information

## Figures and Tables

**Table 1:** Summary of the thermodynamic folding characteristics of mitochondrial pre-mRNAs in their free and editosome-bound RNA folding state.

		pre-mRNA				
		RPS12_pe_	ND3_pe_	A6_pe_	CYb_me_	COI_ne_
*In silico*	ΔG_RNAStructure_	−102	−98	−98	−344	−468
	ΔG_ViennaRNA_	−103	−107	−107	−336	−459
RNA_free_	ΔG_SHAPE_	−152	−137	−137	−496	−823
	ΔG/nt	−0.45	−0.37	−0.34	−0.41	−0.48
	ΔG/bp	−1.46	−1.35	−1.59	−1.27	−1.47
	r(nt_bp_/nt_ss_)	0.62	0.57	0.47	0.64	0.66
RNA_ed.-bound_	ΔG_SHAPE_	−127	−124	−113	−407	−725
	ΔG/nt	−0.38	−0.33	−0.28	−0.33	−0.43
	ΔG/bp	−1.39	−1.23	−1.62	−1.09	−1.39
	r(nt_bp_/nt_ss_)	0.54	0.55	0.37	0.61	0.61
	ΔΔG	25	14	23	89	98
	ΔΔG/nt	0.08	0.04	0.06	0.07	0.06
	ΔΔG/bp	0.07	0.12	−0.03	0.17	0.08
	Δnt_basepaired_ (%)	−7.7	−2.7	−10.1	−3.1	−4.5
	bp preserved (%)	84	82	50	55	52

∆G-values are in kcal/mol. pe: pan-edited, me: marginally edited, ne: never edited.

## References

[b1] Herschlag D. RNA chaperones and the RNA folding problem. J. Biol. Chem. 270, 20871–20874 (1995). 7545662 10.1074/jbc.270.36.20871

[b2] Rajkowitsch L. *et al.* RNA chaperones, RNA annealers and RNA helicases. RNA Biol. 4, 118–130 (2007). 18347437 10.4161/rna.4.3.5445

[b3] Woodson S. A. Taming free energy landscapes with RNA chaperones. RNA Biol. 7, 677–686 (2010). 21045544 10.4161/rna.7.6.13615 PMC3073327

[b4] Grohman J. K. *et al.* A guanosine-centric mechanism for RNA chaperone function. Science 340, 190–195 (2013). 23470731 10.1126/science.1230715 PMC4338410

[b5] Russell R. RNA misfolding and the action of chaperones. Front. Biosci. 13, 1–20 (2008). 17981525 10.2741/2557 PMC2610265

[b6] Aphasizhev R. & Aphasizheva I. Mitochondrial RNA editing in trypanosomes: small RNAs in control. Biochimie 100, 125–131 (2014). 24440637 10.1016/j.biochi.2014.01.003 PMC4737708

[b7] Göringer H. U. ‘Gestalt,’ composition and function of the *Trypanosoma brucei* editosome. Annu Rev Microbiol. 66, 65–82 (2012). 22994488 10.1146/annurev-micro-092611-150150

[b8] Müller U. F. & Göringer H. U. Mechanism of the gBP21-mediated RNA/RNA annealing reaction: matchmaking and charge reduction. Nucleic Acids Res. 30, 447–455 (2002). 11788706 10.1093/nar/30.2.447 PMC99830

[b9] Missel A., Souza A. E., Nörskau G. & Göringer H. U. Disruption of a gene encoding a novel mitochondrial DEAD-box protein in *Trypanosoma brucei* affects edited mRNAs. Mol. Cell. Biol. 17, 4895–4903 (1997). 9271369 10.1128/mcb.17.9.4895 PMC232342

[b10] Li F., Herrera J., Zhou S., Maslov D. A. & Simpson L. Trypanosome REH1 is an RNA helicase involved with the 3′-5′ polarity of multiple gRNA-guided uridine insertion/deletion RNA editing. Proc. Natl. Acad. Sci. USA. 108, 3542–3547 (2011). 21321231 10.1073/pnas.1014152108 PMC3048136

[b11] Kruse E., Voigt C., Leeder W.-M. & Göringer H. U. RNA helicases involved in U-insertion/deletion-type RNA editing. Biochim. Biophys. Acta 1829, 835–841 (2013). 23587716 10.1016/j.bbagrm.2013.04.003

[b12] Böhm C., Katari V. S., Brecht M. & Göringer H. U. *Trypanosoma brucei* 20S editosomes have one RNA substrate-binding site and execute RNA unwinding activity. J. Biol. Chem. 287, 26268–26277 (2012). 22661715 10.1074/jbc.M112.365916 PMC3406711

[b13] Merino E. J., Wilkinson K. A., Coughlan J. L. & Weeks K. M. RNA structure analysis at single nucleotide resolution by selective 2′-hydroxyl acylation and primer extension (SHAPE). J. Am. Chem. Soc. 127, 4223–4231 (2005). 15783204 10.1021/ja043822v

[b14] Low J. T. & Weeks K. M. SHAPE-directed RNA secondary structure prediction. Methods 52, 150–158 (2010). 20554050 10.1016/j.ymeth.2010.06.007 PMC2941709

[b15] Weeks K. M. Toward all RNA structures, concisely. Biopolymers 103, 438–448 (2015). 25546503 10.1002/bip.22601 PMC4446244

[b16] Mortimer S. A. & Weeks K. M. A fast-acting reagent for accurate analysis of RNA secondary and tertiary structure by SHAPE chemistry. J. Am. Chem. Soc. 129, 4144–4145 (2007). 17367143 10.1021/ja0704028

[b17] McGinnis J. L., Dunkle J. A., Cate J. H. & Weeks K. M. The mechanisms of RNA SHAPE chemistry. J. Am. Chem. Soc. 134, 6617–6624 (2012). 22475022 10.1021/ja2104075 PMC4337229

[b18] Hofacker I. L. Vienna RNA secondary structure server. Nucleic Acids Res. 31, 3429–3431 (2003). 12824340 10.1093/nar/gkg599 PMC169005

[b19] Reuter J. S. & Mathews D. H. RNAstructure: software for RNA secondary structure prediction and analysis. BMC Bioinformatics 11, 129 (2010). 20230624 10.1186/1471-2105-11-129 PMC2984261

[b20] Millevoi S., Moine H. & Vagner S. G-quadruplexes in RNA biology. Wiley Interdiscip. Rev. RNA 3, 495–507 (2012). 22488917 10.1002/wrna.1113

[b21] Shrestha P., Xiao S., Dhakal S., Tan Z. & Mao H. Nascent RNA transcripts facilitate the formation of G-quadruplexes. Nucleic Acids Res. 42, 7236–7246 (2014). 24829453 10.1093/nar/gku416 PMC4066803

[b22] Mergny J. L., Phan A. T. & Lacroix L. Following G-quartet formation by UV-spectroscopy. FEBS Lett. 435, 74–78 (1998). 9755862 10.1016/s0014-5793(98)01043-6

[b23] Mergny J. L., Li J., Lacroix L., Amrane S. & Chaires J. B. Thermal difference spectra: a specific signature for nucleic acid structures. Nucleic Acids Res. 33, e138 (2005). 16157860 10.1093/nar/gni134 PMC1201377

[b24] Zheng K. W. *et al.* A competitive formation of DNA:RNA hybrid G-quadruplex is responsible to the mitochondrial transcription termination at the DNA replication priming site. Nucleic Acids Res. 42, 10832–10844 (2014). 25140009 10.1093/nar/gku764 PMC4176368

[b25] Wu R. Y., Zheng K. W., Zhang J. Y., Hao Y. H. & Tan Z. Formation of DNA:RNA hybrid G-quadruplex in bacterial cells and its dominance over the intramolecular DNA G-quadruplex in mediating transcription termination. Angew. Chem. Int. Ed. Engl. 54, 2447–2451 (2015). 25613367 10.1002/anie.201408719

[b26] Hajdin C. E. *et al.* Accurate SHAPE-directed RNA secondary structure modeling, including pseudoknots. Proc. Natl. Acad. Sci. USA 110, 5498–5503 (2013). 23503844 10.1073/pnas.1219988110 PMC3619282

[b27] Vendeix F. A., Munoz A. M. & Agris P. F. Free energy calculation of modified base-pair formation in explicit solvent: A predictive model. RNA 15, 2278–2287 (2009). 19861423 10.1261/rna.1734309 PMC2779691

[b28] Lorentzen E., Basquin J., Tomecki R., Dziembowski A. & Conti E. Structure of the active subunit of the yeast exosome core, Rrp44: diverse modes of substrate recruitment in the RNase II nuclease family. Mol. Cell 29, 717–728 (2008). 18374646 10.1016/j.molcel.2008.02.018

[b29] Bonneau F., Basquin J., Ebert J., Lorentzen E. & Conti E. The yeast exosome functions as a macromolecular cage to channel RNA substrates for degradation. Cell 139, 547–559 (2009). 19879841 10.1016/j.cell.2009.08.042

[b30] Czerwoniec A., Kasprzak J. M., Bytner P., Dobrychłop M. & Bujnicki J. M. Structure and intrinsic disorder of the proteins of the *Trypanosoma brucei* editosome. FEBS Lett. 589, 2603–2610 (2015). 26226426 10.1016/j.febslet.2015.07.026

[b31] Tompa P. & Csermely P. The role of structural disorder in the function of RNA and protein chaperones. FASEB J. 18, 1169–1175 (2004). 15284216 10.1096/fj.04-1584rev

[b32] Dethoff E. A., Chugh J., Mustoe A. M. & Al-Hashimi H. M. Functional complexity and regulation through RNA dynamics. Nature 482, 322–330 (2012). 22337051 10.1038/nature10885 PMC3320162

[b33] Bokinsky G. *et al.* Two distinct binding modes of a protein cofactor with its target RNA. J. Mol. Biol. 361, 771–784 (2006). 16872630 10.1016/j.jmb.2006.06.048 PMC2633024

[b34] Shajani Z., Drobny G. & Varani G. Binding of U1A protein changes RNA dynamics as observed by ^13^C NMR relaxation studies. Biochemistry 46, 5875–5883 (2007). 17469848 10.1021/bi602658x

[b35] Kim H. *et al.* Protein-guided RNA dynamics during early ribosome assembly. Nature 506, 334–338 (2014). 24522531 10.1038/nature13039 PMC3968076

[b36] Cross G. A. Identification, purification and properties of clone specific glycoprotein antigens constituting the surface coat of *Trypanosoma brucei*. Parasit. 71, 393–417 (1975). 10.1017/s003118200004717x 645

[b37] Brun R. & Schönenberger M. Cultivation and *in vitro* cloning or procyclic culture forms of *Trypanosoma brucei* in a semi-defined medium. Acta Tropica 36, 289–292 (1979). 43092

[b38] Hauser R., Pypaer M., Häusler T., Horn E. K. & Schneider A. *In vitro* import of proteins into mitochondria of *Trypanosoma brucei* and *Leishmania tarentolae*. J. Cell Sci. 109, 517–523 (1996). 8838675 10.1242/jcs.109.2.517

[b39] Göringer H. U., Koslowsky D. J., Morales T. H. & Stuart K. The formation of mitochondrial ribonucleoprotein complexes involving guide RNA molecules in *Trypanosoma brucei*. Proc. Natl. Acad. Sci. USA 91, 1776–1780 (1994). 8127880 10.1073/pnas.91.5.1776 PMC43246

[b40] Igo R. P. Jr., Palazzo S. S., Burgess M. L., Panigrahi A. K. & Stuart K. Uridylate addition and RNA ligation contribute to the specificity of kinetoplastid insertion RNA editing. Mol. Cell. Biol. 20, 8447–8457 (2000). 11046141 10.1128/mcb.20.22.8447-8457.2000 PMC102151

[b41] Igo R. P. Jr. *et al.* Role of uridylate-specific exoribonuclease activity in *Trypanosoma brucei* RNA editing. *Eukar*. Cell 1, 112–118 (2002). 10.1128/EC.1.1.112-118.2002 PMC118049 12455977

[b42] Katari V. S., van Esdonk L. & Göringer H. U. Molecular crowding inhibits U-insertion/deletion RNA editing *in vitro*: consequences for the *in vivo* reaction. PLoS One 8, e83796 (2013). 24376749 10.1371/journal.pone.0083796 PMC3871654

[b43] Turner R., Shefer K. & Ares M. Jr. Safer one-pot synthesis of the ‘SHAPE’ reagent 1-methyl-7-nitroisatoic anhydride (1m7). RNA 19, 1857–1863 (2013). 24141619 10.1261/rna.042374.113 PMC3884660

[b44] Vasa S. M., Guex N., Wilkinson K. A., Weeks K. M. & Giddings M. C. ShapeFinder: a software system for high-throughput quantitative analysis of nucleic acid reactivity information resolved by capillary electrophoresis. RNA 14, 1979–1990 (2008). 18772246 10.1261/rna.1166808 PMC2553743

[b45] Deigan K. E., Li T. W., Mathews D. H. & Weeks K. M. Accurate SHAPE-directed RNA structure determination. Proc. Natl. Acad. Sci. USA. 106, 97–102 (2009). 19109441 10.1073/pnas.0806929106 PMC2629221

[b46] Lorenz R. *et al.* 2D meets 4 G: G-quadruplexes in RNA secondary structure prediction. IEEE/ACM Trans. Comput. Biol. Bioinform. 10, 832–844 (2013). 24334379 10.1109/TCBB.2013.7

[b47] Savitzky A. & Golay M. J. E. Smoothing and differentiation of data by simplified least squares procedures. Anal. Chem. 36, 1627–1639 (1964).

[b48] Kibbe W. A. OligoCalc: an online oligonucleotide properties calculator. Nucleic Acids Res. 35 (Web Server issue), W43–46 (2007). 17452344 10.1093/nar/gkm234 PMC1933198

