# Supplementary material for: The RNA chaperone activity of the Trypanosoma brucei editosome raises the dynamic of bound pre-mRNAs
Source: Sci Rep. 2016 Jan 19;6:19309. doi: 10.1038/srep19309 (PMC4726059; doi:10.1038/srep19309)

The RNA chaperone activity of the *Trypanosoma brucei* editosome raises the dynamic of bound pre-mRNAs

W.-Matthias Leeder, Christin Voigt, Michael Brecht & H. Ulrich Göringer

Molecular Genetics, Darmstadt University of Technology, Schnittspahnstraße 10, 64287 Darmstadt, Germany

Correspondence and requests for materials should be addressed to HUG (goringer@bio.tu-darmstadt.de).

Supplementary Tables /Figures

Supplementary Table. 1. Nucleotide composition of the *T. brucei* mitochondrial pre-mRNAs used in this study.

	pre-mRNA				
	$RPS12_{pe}$	$ND3_{pe}$	$A6_{pe}$	CYb_{me}	COI_{ne}
length (nt)	282	322	344	1080	1647
A	85	110	117	327	372
U	93	100	75	505	788
G	75	86	132	179	285
C	29	26	20	69	202
R/Y	1.3	1.6	2.6	0.9	0.7

pe: pan-edited, me: marginally edited, ne: never edited.

Supplementary Table. 2. Summary of the identified 2D-structural features of the 5 *T. brucei* mitochondrial pre-mRNAs in their free RNA folding states.

	pre-mRNA				
	$RPS12_{pe}$	$ND3_{pe}$	$A6_{pe}$	CYb_{me}	COI_{ne}
longest ds	17	10	9	17	15
longest ss	9	8	18	17	13
GU bp	21	15	17	71	107
other non-canonical bp	1	0	0	12	29
G-quadruplex	0	2	4	0	0
pseudoknots	1	0	0	0	0
hairpin loops	8	9	7	24	36
tri/tetra loops	3	5	2	7	14
internal loops	7	6	2	25	23
symmetric	2	2	1	8	8
asymmetric	5	4	1	17	15
external loops	1	1	1	1	1
multi-loops	2	2	3	8	11
bulges	2	4	3	12	26

pe: pan-edited, me: marginally edited, ne: never edited.

Supplementary Figure 1. Experimental variation of SHAPE-modification experiments. (**A**) Representative Pearson plot of SHAPE-reactivity data of an experimental replicate (exp. 1, exp. 2) of the RPS12 transcript. (**B**) Representative Pearson plot of a biochemical replicate (prep. 1, prep. 2) of the RPS12 pre-mRNA. (**C**) Summary of Pearson correlation coefficients (*r*) and number of experimental replicates for all *T. brucei* transcripts in their free and editosome-bound (ed.-bound) folding states.

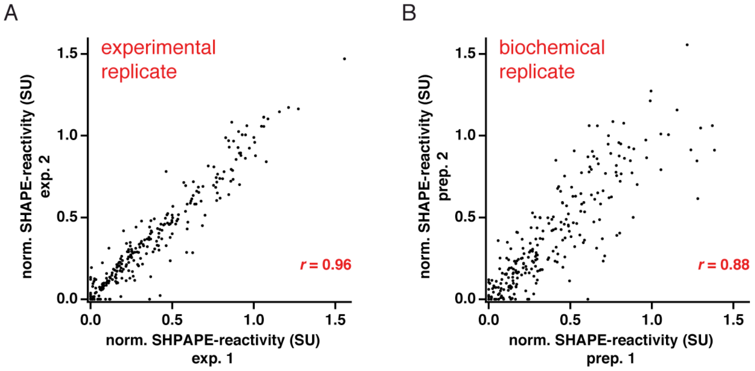

C

pre-mRNA	pearson (*r*) ±SD	replicates
$RPS12_{free}$	0.87 ± 0.04	5
$RPS12_{ed.\text{-}bound}$	0.81 ± 0.07	3
$ND3_{free}$	0.86 ± 0.06	4
$ND3_{ed.\text{-}bound}$	0.73 ± 0.10	7
$A6_{free}$	0.83 ± 0.07	5
$A6_{ed.\text{-}bound}$	0.79 ± 0.09	4
CYb_{free}	0.82 ± 0.06	8
$CYb_{ed.\text{-}bound}$	0.79 ± 0.07	3
COI_{free}	0.83 ± 0.05	3
$COI_{ed.\text{-}bound}$	0.60 ± 0.05	4

Supplementary Figure 2. Workflow to generate normalized SHAPE-profiles. Capillary electrophoresis (CE)-trace of the RPS12 transcript in its free folding state ($RPS12_{free}$) followed by all operational steps to generate normalized SHAPE-modification profiles. RFU: relative fluorescence unit.

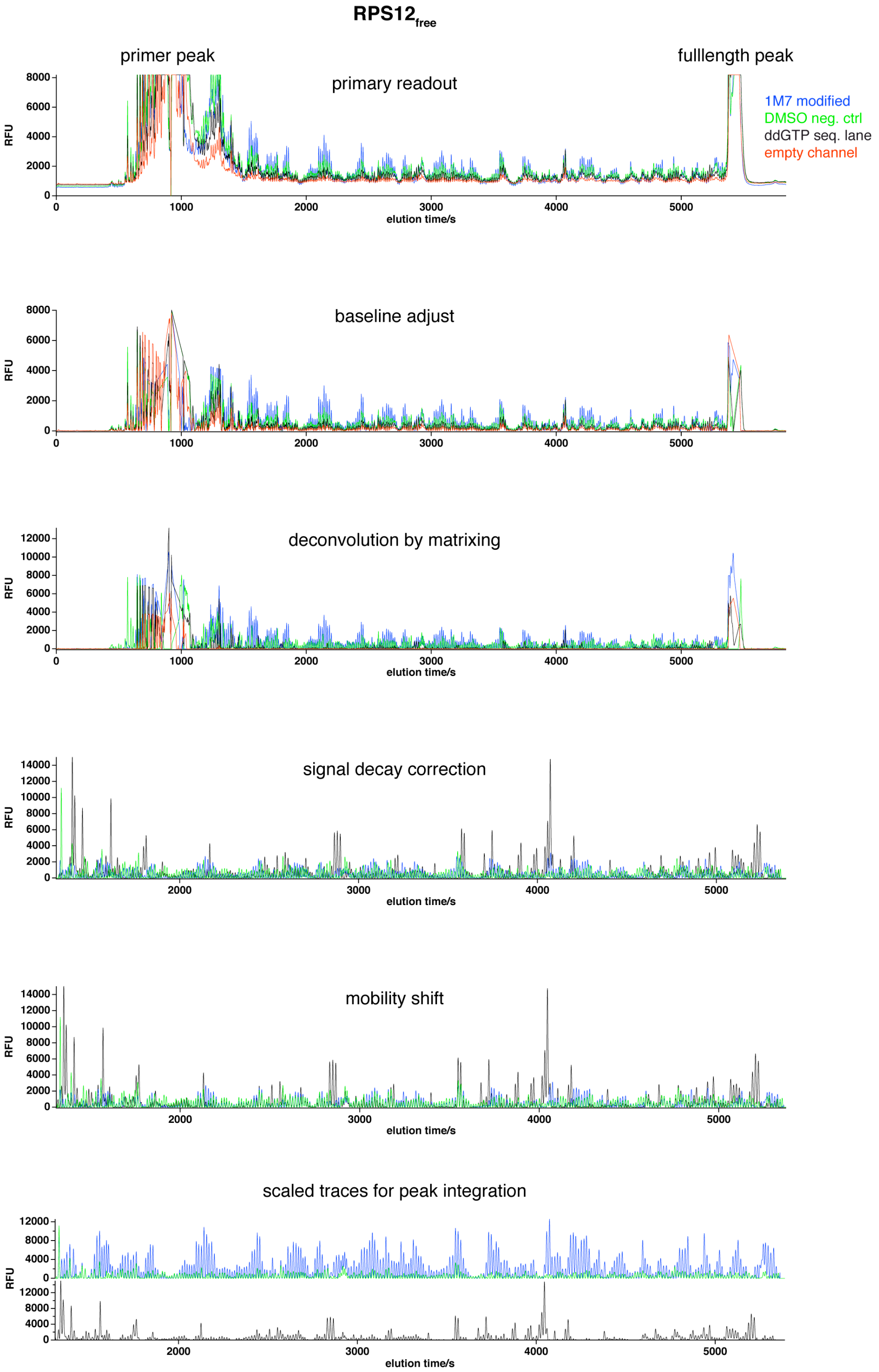

Supplementary Figure 3. UV-melting curves. Fraction folded (α) *versus* temperature plots (solid lines) and normalized 1st-derivatives (dashed lines) of the never-edited COI-RNA and the pre-edited CYb-, RPS12-, ND3- and A6-transcripts in their free folding states. Colors annotate the percentage of unfolded RNA (see legend). All transcripts show complex melting profiles with a main melting transition around 50°C and additional helix/coil transitions ≥70°C (A6, ND3, RPS12). Bottom table: Comparison of experimentally- and theoretically-derived hyperchromicities ($A_{rel.}$) (see Methods section). Errors are standard deviations (SD).

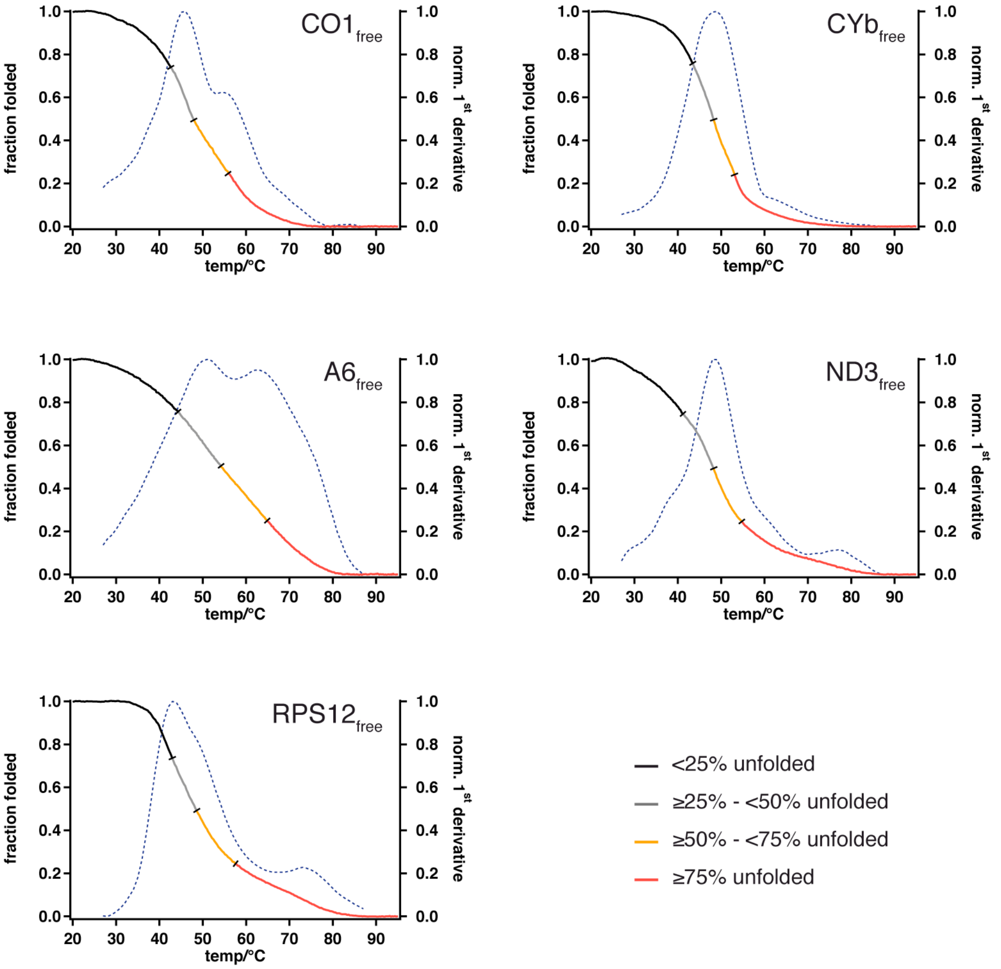

	measured hyperchromicity (%)	predicted hyperchromicity (%)	
pre-mRNA	UV-melting curves	"nearest neighbour"	"fractional approach"
COI_{free}	20.3±1.0	30	22
Cyb_{free}	31.5±1.6	32	21
$A6_{free}$	20.3±1.0	19	15
$ND3_{free}$	17.2±0.9	20	16
$RPS12_{free}$	20.5±1.2	25	20

Supplementary Figure 4. SHAPE-reactivity profiles of *T. brucei* mitochondrial pre-mRNAs in their editosome-bound folding state. Normalized SHAPE-reactivity profiles of all 5 transcripts in their editosome-bound folding state (ed.-bound). Black: low (≤0.35SU); yellow: medium (0.35<SU≤0.8); red: high (>0.8SU) normalized SHAPE-reactivities. SU: SHAPE-unit. nt: nucleotides.

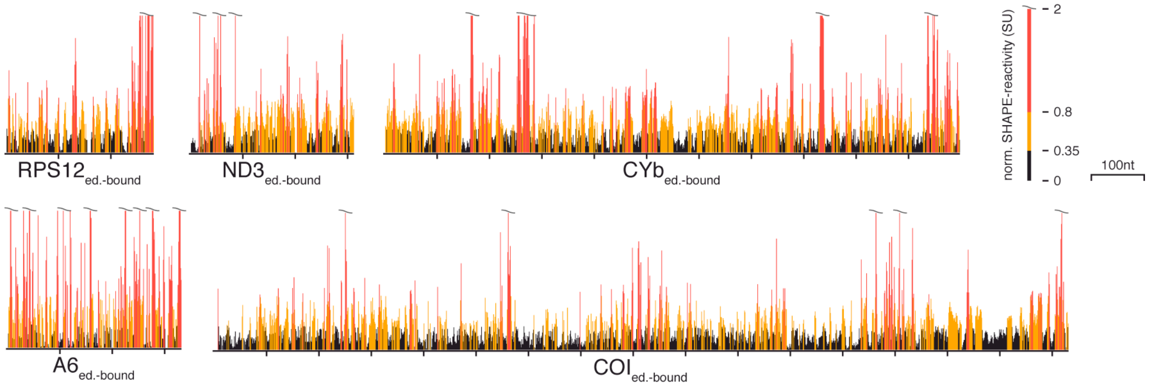

Supplementary Figure 5. Summary of SHAPE-reactivities in the free and editosome-bound RNA folding state. Histograms comparing the normalized SHAPE-reactivities of all 5 *T. brucei* mitochondrial transcripts in their free and editosome-bound (ed.-bound) folding state. SHAPE-reactivities were binned in 0.1 SHAPE-units (SU) and plotted as stacked bars in percent. The median SHAPE-reactivity is shown in italics. The arithmetic mean (AM) is marked as a black line. Editosome binding increases the AM of all transcripts especially the 3 pan-edited pre-mRNAs (A6, ND3, RPS12) indicating an increase in structural flexibility.

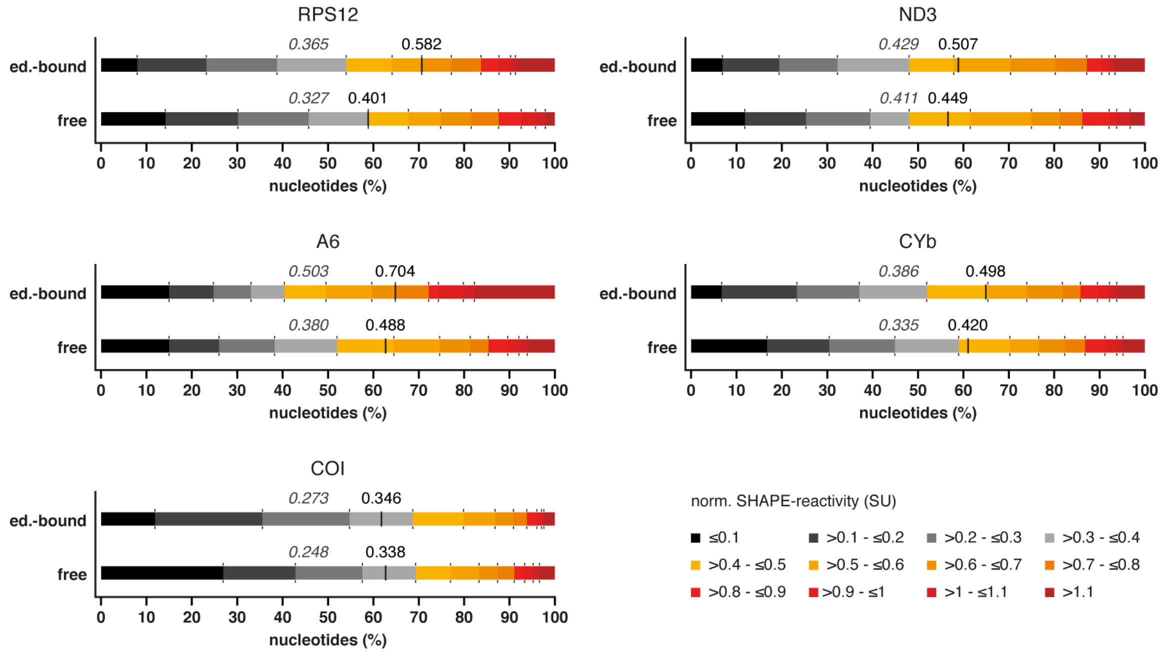

Supplementary Figure 6. Nucleotide specificity of the free to editosome-bound RNA-folding transition. For each pre-mRNA transcript, the number of affected nt was set to 100% and divided into 4 classes: nucleotides that become slightly more flexible (ΔSHAPE 0.2≤SU<0.35 - light green), significantly more flexible (ΔSHAPE ≥0.35SU - dark green), slightly less flexible (ΔSHAPE -0.2≥SU>-0.35 - cyan) and significantly less flexible (ΔSHAPE ≤-0.35 - dark blue) in the editosome-bound RNA folding state. A change of 0.35 in either direction can represent the transition from a bp- to a ss-conformation or *vice versa*. Data were plotted as stacked bars in percent. Black lines: relative abundance of the different nucleotides in the various transcripts.

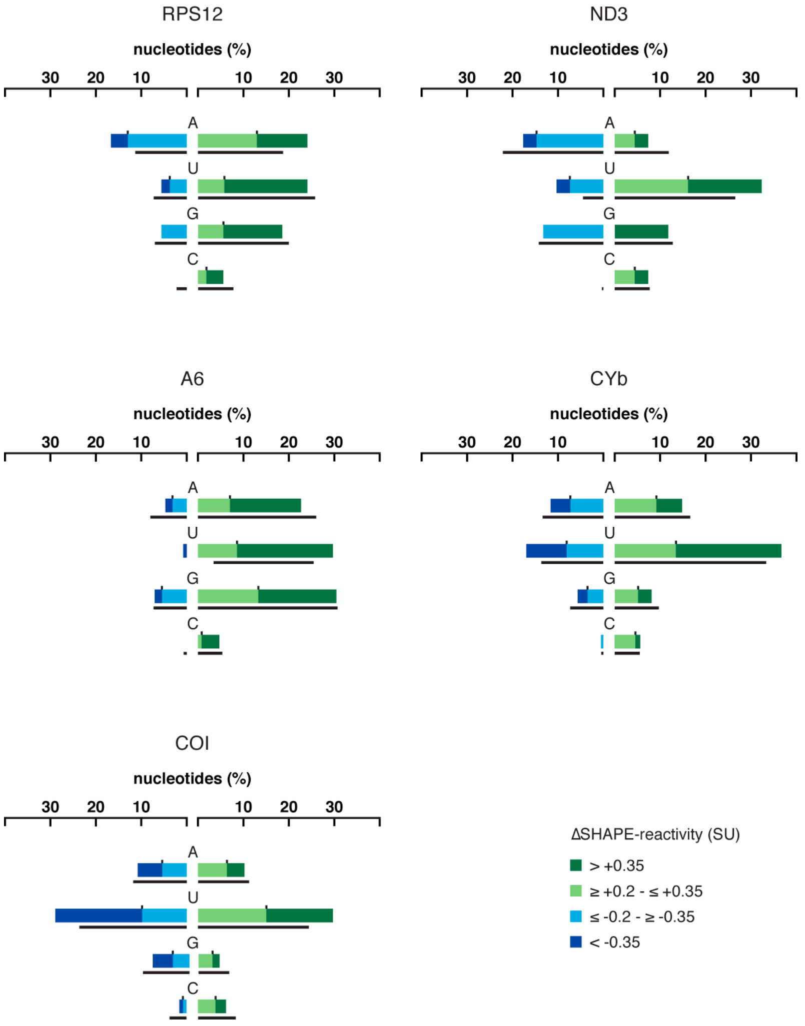

Supplementary Figure 7. *In vitro* RNA editing. Pre-cleaved *in vitro* U-insertion (A) and U-deletion (B) RNA editing assays to determine the editing activity of twelve *T. brucei* mitochondrial fractions (F1-12). The U-insertion assay monitors the insertion of 3 U nucleotides; the U-deletion reaction the removal of 4 U's. RNA reactants, intermediates and edited products (annotated to the right of the gels) were electrophoretically separated and densitometrically quantified. *: position of the radioactive label (^{32}P). Arrows indicate the position of the fully edited mRNA products. Fractions numbered in green were used for the SHAPE-experiments in this study.

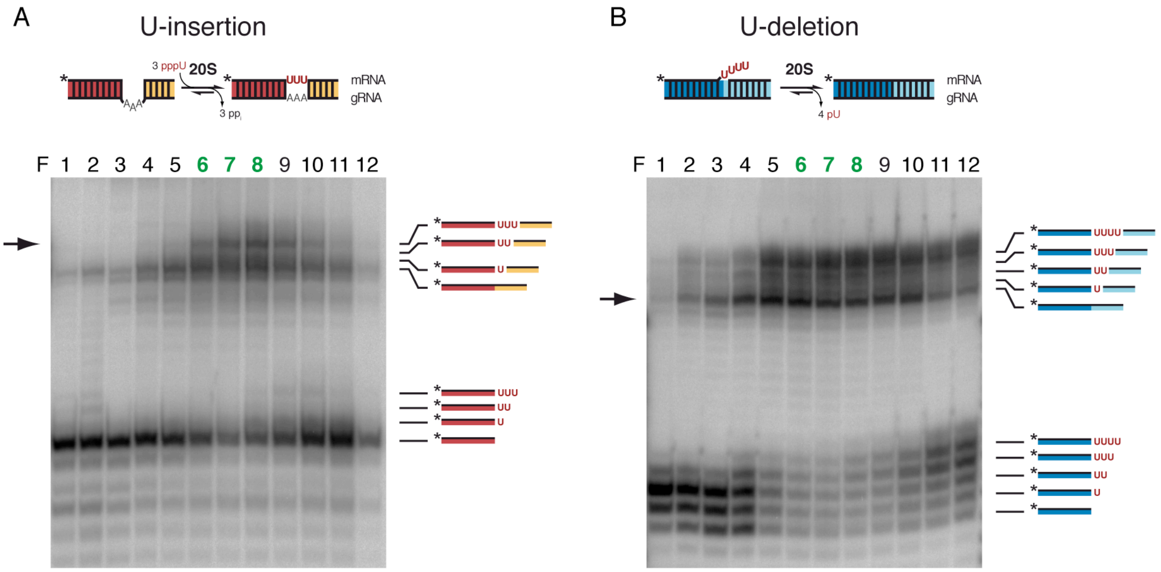

Supplementary Figure 8. “gDNA”/pre-mRNA-hybrid formation in the free and editosome-bound RNA folding states. The figure illustrates an example of the RNaseH-experiment and demonstrates all steps to derive quantitative data: (A) initial phosphorimaging output, raw densitometry traces, offset correction, background subtraction/normalization and (B) peak integration.

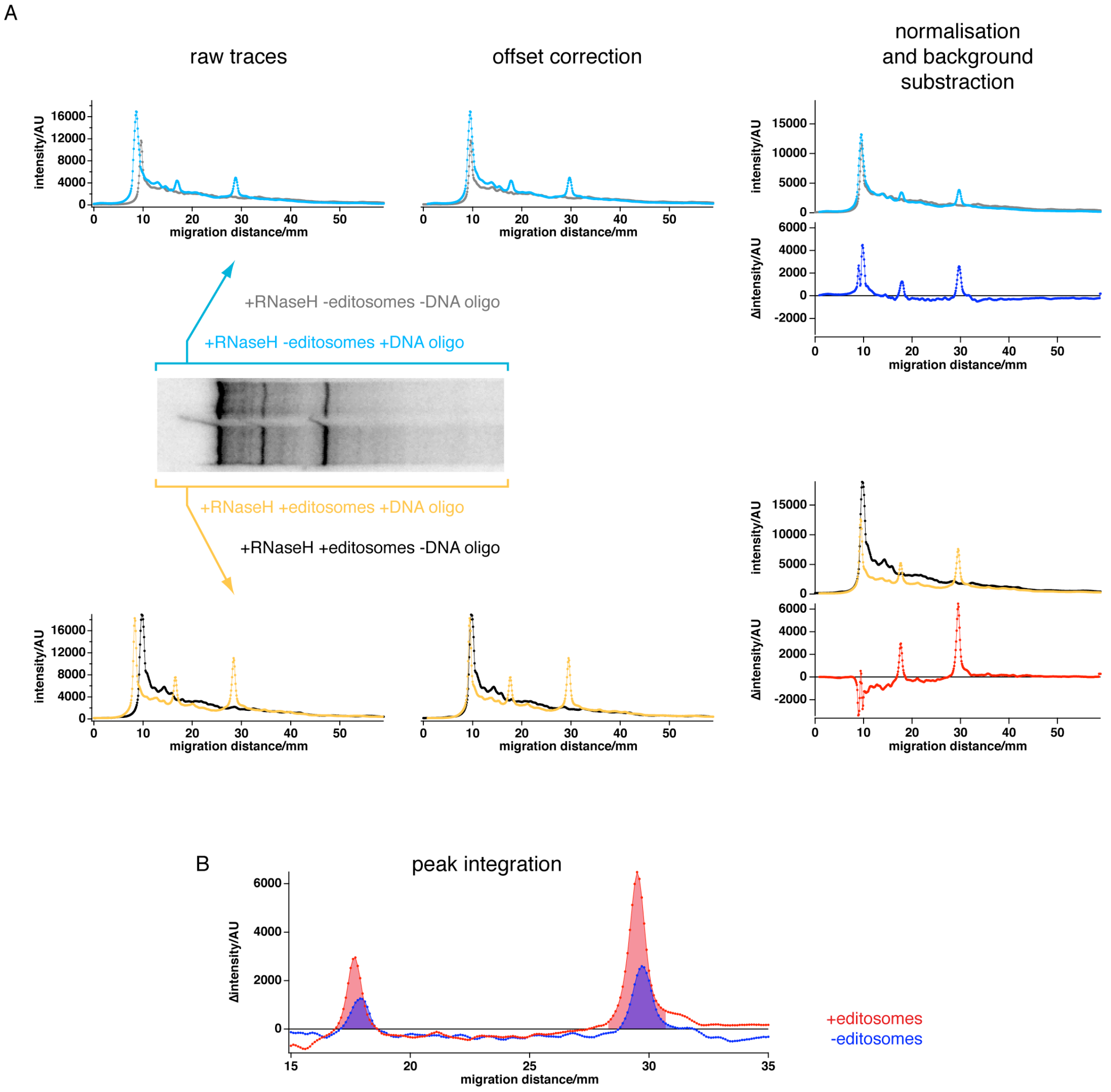

Supplement: Supplementary Information [file srep19309-s1.pdf]
